# Curcumin suppresses tumor necrosis factor-α-induced matrix metalloproteinase-2 expression and activity in rat vascular smooth muscle cells via the NF-κB pathway

**DOI:** 10.3892/etm.2014.1647

**Published:** 2014-03-28

**Authors:** YI ZHONG, WENYAN YU, JIAN FENG, ZHONGCAI FAN, JIAFU LI

**Affiliations:** 1 Department of Cardiology, The Affiliated Hospital of Luzhou Medical College, Luzhou, Sichuan 646000, P.R. China; 2 Department of Pathophysiology, Southern Medical University, Guangzhou, Guangdong 510515, P.R. China

**Keywords:** curcumin, tumor necrosis factor-α, nuclear factor-κB, matrix metalloproteinase-2

## Abstract

The aim of the present study was to investigate the inhibitory effect of curcumin on tumor necrosis factor (TNF)-α-induced cell migration and matrix metalloproteinase (MMP)-2 expression and activity in rat vascular smooth muscle cells (VSMCs), in order to identify whether the effects are mediated by the nuclear factor (NF)-κB signaling pathway. The VSMCs cells were pretreated with curcumin prior to stimulation with TNF-α. Reverse transcription-polymerase chain reaction and western blot analysis were used to determine the MMP-2 mRNA and protein expression levels in TNF-α-stimulated VSMCs. Activity levels of MMP-2 were measured using a gelatin zymography assay. Intracellular reactive oxygen species (ROS) generation was also analyzed. Curcumin was found to suppress the TNF-α-stimulated migration of VSMCs. In addition, curcumin inhibited the TNF-α-induced induction of MMP-2 activity and expression. Curcumin also decreased ROS generation in TNF-α-stimulated VSMCs. Signal transduction experiments indicated that TNF-α-induced MMP-2 expression in VSMCs was partly reversed with the application of an NF-κB inhibitor (BAY11-7082). In addition, western blot analysis revealed that curcumin reduced NF-κB p65 protein expression in TNF-α-stimulated VSMCs at the concentration of 20 and 40 μM. Therefore, these observations indicated that curcumin suppressed TNF-α-stimulated VSMC migration and partially prevented TNF-α-induced MMP-2 expression and activity in VSMCs via the NF-κB pathway.

## Introduction

The proliferation and migration of vascular smooth muscle cells (VSMCs) may play a key role in the development of intimal thickening following arterial-wall injury or in atherosclerosis (1). Matrix metalloproteinases (MMPs) are a broad family of zinc-dependent proteinases, which are implicated in extracellular matrix turnover, vascular wall remodeling, angiogenesis and atherosclerosis (2). Among the MMP family, several lines of evidence have hypothesized that MMP-2 plays a key role in promoting VSMC proliferation, migration and the weakening of atherosclerotic plaque stability (3). In addition, MMP-2 expression in VSMCs has been associated with a variety of pathological situations, particularly in atherosclerotic plaques, which show significantly increased MMP-2 expression and activation levels, most prominently in vulnerable regions, indicating a pathogenic role for MMP-2 in the progression of atherosclerosis (4,5). Curcumin (diferuloylmethane), a bioactive constituent from *Curcuma longa,* possesses marked anti-inflammatory, antioxidant and anticarcinogenic properties (6,7). A previous study has shown that curcumin possesses anti-inflammatory, antioxidant, anticancer, antibacterial and antiviral activities, and exhibits a strong potency in inhibiting transcription factors, protein kinases, cytokines, adhesion molecules and oxidative stress (8). In the present study, the inhibitory effect of curcumin on tumor necrosis factor (TNF)-α-induced VSMC migration and MMP-2 expression and activity was investigated, with the aim of identifying the pathways involved.

## Materials and methods

### Cell culture and treatment

Rat aortic smooth muscle cells were isolated from male Sprague-Dawley rats (purchased from the laboratory animal center of Southern Medical University, Guangzhou, China), as described previously (9). The cells were cultured in Dulbecco’s modified Eagle’s medium (Gibco-BRL, Carlsbad, CA, USA) supplemented with 10% fetal bovine serum (Gibco-BRL), 25 mM HEPES, 100 U/ml penicillin and 100 μg/ml streptomycin at 37°C. Cells that had grown to 80–90% confluence were used for all the experiments. Cells were placed in serum-free medium for 24 h prior to treatment with TNF-α (Sigma, St. Louis, MO, USA). The study was conducted in strict accordance with the recommendations in the Guide for the Care and Use of Laboratory Animals of the National Institutes of Health. Prior to the addition of TNF-α to the medium, the cells were pretreated with curcumin (Solon, OH, USA). The study was approved by the Ethics Committee of Southern Medical University.

### Cell viability assay

Cells were plated with a variety of concentrations of curcumin (0–40 μM) in 96-well microtiter plates, and were then cultured for 24 h at 37°C in a 5% CO_2_ incubator. Cell viability was determined using the conventional methylthiazolyl tetrazolium (MTT) reduction assay. Following the treatment of the cells with curcumin, MTT solution was added (final concentration, 5 mg/ml) and incubation was continued for 4 h at 37°C. The dark blue formazan crystals formed in the intact cells were solubilized with dimethyl sulfoxide and the absorbance of the blue color was measured at 490 nm using a microplate reader.

### Preparations of nuclear proteins

Cells (1×10^7^ cells/ml) were harvested, washed with ice-cold phosphate-buffered saline (PBS), centrifuged and resuspended in ice-cold isotonic buffer A [10 mM HEPES (pH 7.9), 10 mM KCl, 1.5 mM MgCl_2_, 0.5 mM dithiothreitol (DTT) and 0.5 mM phenylmethanesulfonyl fluoride (PMSF)]. Following incubation in an ice bath for 15 min, the cells were centrifuged at 16,000 × g for 5 min at 4°C. The cells were then resuspended in ice-cold buffer C [20 mM HEPES (pH 7.9), 20% glycerol, 0.4 M NaCl, 1.5 mM MgCl_2_, 0.2 mM EDTA, 0.5 mM DTT and 0.5 mM PMSF], which was followed by incubation at 4°C for 40 min. Following vortex-mixing, the resulting suspension was centrifuged at 16,000 × g for 10 min at 4°C, and the supernatant was stored at −80°C. The protein content was determined by bicinchoninic protein assay reagent.

### Reverse transcription-polymerase chain reaction (RT-PCR)

Total RNA was isolated from cells using TRIzol-reagent (Invitrogen Life Technologies, Carlsbad, CA, USA) and quantified by ultraviolet absorption at 260 and 280 nm. RT-PCR was performed according to the manufacturer’s instructions. According to GenBank, the RT-PCR primers were designed as follows: MMP-2 sense, 5′-ACCTGTCACTCCGGAGATCTGCAA-3′ and antisense, 5′-TCACGCTCTTGAGACTTTGGTTCT-3′. The PCR conditions were as follows: 30 cycles of 94°C for 30 sec; 55°C for 30 sec; and 72°C for 45 sec. The amplified products were visualized by 1.5% agarose gel electrophoresis, stained with ethidium bromide and images were then captured under ultraviolet light. Densitometric analysis of the different observations was performed using Quantity One Software (Bio-Rad, Hercules, CA, USA). The quantity of each transcript was normalized against GAPDH.

### Western blot analysis

VSMCs were harvested at the indicated time points and lysed into lysis buffer. Total proteins (50 μg per well) were separated by 10% SDS-PAGE and electrophoretically transferred to nitrocellulose membranes. Following blocking for 1 h with 5% skimmed milk in Tris-buffered saline (TBS; 10 mM Tris and 150 mM NaCl), the membrane was washed three times for 15 min each with TBS Tween-20 buffer (10 mM Tris, 150 mM NaCl and 0.1% Tween-20). Immunoreactive bands were visualized using horseradish peroxidase-conjugated secondary antibodies and an enhanced chemiluminescence (ECL) western blotting detection kit (GE Healthcare, Little Chalfont, UK). The bands were visualized using the ECL system, and the band density was determined using Image J software. All the antibodies were purchased from the Beyotime Institute of Biotechnology (Shanghai, China).

### Gelatin zymography

Enzymatic activity levels of MMP-2 were assessed by gel zymographic analysis (10). The protein content of the samples was measured by the colorimetric method using serum albumin as the standard. In total, 50-μg samples of proteins from the VSMC lysate were loaded on an 11% SDS-PAGE gel containing 0.1% gelatin for electrophoresis in a 4°C cold room. Subsequently, the gels were incubated with collagenase buffer for 16 h at 37°C, stained with 0.25% Coomassie Brilliant Blue, destained with 30% isopropanol in 10% acetic acid and visualized.

### Cell migration assay

The invasion of VSMCs through the extracellular matrix was determined using a commercial cell invasion assay kit (Chemicon International, Temecula, CA, USA), as described in a previous study (11). VSMCs were resuspended in conditioned medium that had been collected following pretreatment with curcumin and TNF-α-treated cells for 23 h, and were added to the upper components of the migration chamber. Next, a 500-μl sample of the same conditioned medium was added to the lower compartment of the migration chamber. Cells without TNF-α-treated conditioned medium served as the control. The migration chambers were incubated at 37°C for 24 h in an atmosphere of 5% CO_2_. Following incubation, the inserts were removed from the wells and the cells on the upper side of the filter were removed using cotton swabs. The filters were fixed and stained according to the manufacturer’s instructions. Next, 100 μl dye mixture was transferred to a 96-well plate and the optical density was measured at 560 nm.

### Measurement of reactive oxygen species (ROS)

Prior to chemical treatment, the cells were incubated in culture medium containing 30 μM 2′,7-dichlorofluorescein (DCF; Beyotime Institute of Biotechnology, Shanghai, China), a fluorescent dye, for 30 min to establish a stable intracellular level of the probe. Subsequently, the cells were washed with PBS, removed from the Petri dishes by scraping and evaluated for DCF fluorescence intensity. This was used as an index of the intracellular levels of ROS. The fluorescent DCF was detected using a laser scanning confocal microscope (TCS-NT; Leica Microsystems, Heidelberg, Germany) with excitation and emission wavelengths of 488 and 520 nm, respectively. The cell number in each sample was counted and utilized to normalize the fluorescence intensity of DCF.

### Statistical analysis

Data are expressed as the mean ± standard deviation of three assays. Statistical analysis was conducted using one-way analysis of variance and P<0.05 was considered to indicate a statistically significant difference. All statistical analyses were performed using SPSS 13.0 software (SPSS, Inc., Chicago, IL, USA).

## Results

### Assessment of cell toxicity of curcumin

**Figure 1:**
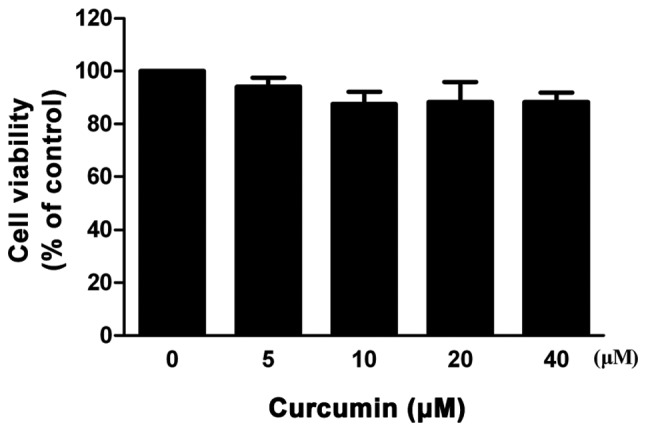
Cell toxicity of curcumin. The cytotoxic effect of curcumin in VSMCs was determined by an MTT assay. Data are expressed as the mean ± standard deviation of three independent experiments. VSMCs, vascular smooth muscle cells; MTT, methylthiazolyl tetrazolium.

The MTT assay was performed to evaluate the cytotoxicity of curcumin on VSMCs. As shown in Fig. 1, curcumin did not exhibit a dose-dependent (0–40 μM) cytotoxic effect in VSMCs. According to the results of the MTT assay, curcumin concentrations of 20 and 40 μM were selected for all the following experiments.

### Curcumin inhibits TNF-α-stimulated migration in VSMCs

**Figure 2:**
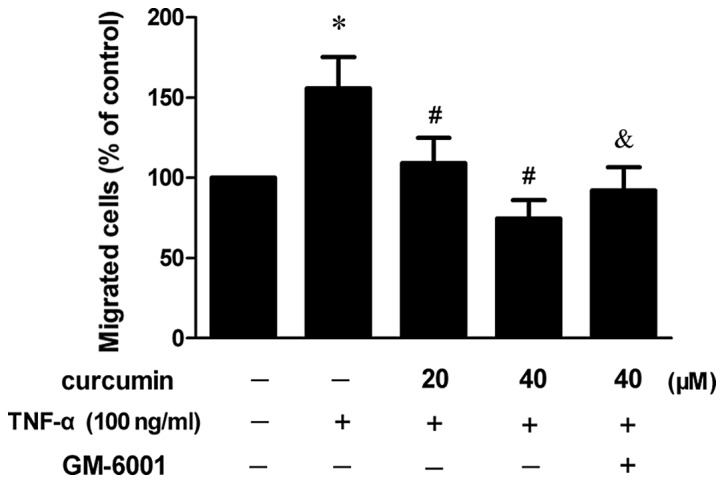
Curcumin inhibits TNF-α-stimulated migration in VSMCs. VSMCs were resuspended in a conditioned medium following treatment with TNF-α for 23 h, and were added to the upper components of the migration chamber in the presence of 20 and 40 μM curcumin (or 10 μM GM-6001). ^*^P<0.05, vs. control group; ^#^P<0.05, vs. TNF-α-treated group; ^&^P<0.05, vs. TNF-α + curcumin group. Data are expressed as the mean ± standard deviation of three independent experiments. VSMCs, vascular smooth muscle cells; TNF, tumor necrosis factor.

To investigate the effect of curcumin on the migration ability of VSMCs, a cell migration assay was performed. As shown in Fig. 2, the migration of VSMCs increased following treatment with 100 ng/ml TNF-α, whereas 20 and 40 μM curcumin reduced cell migration in TNF-α-induced VSMCs and a statistically significant difference was observed (P<0.05). When VSMCs were pretreated with an MMP inhibitor (GM-6001), the inhibitory effect was partly diminished.

### Curcumin inhibits TNF-α-induced MMP-2 expression and activity in VSMCs

**Figure 3:**
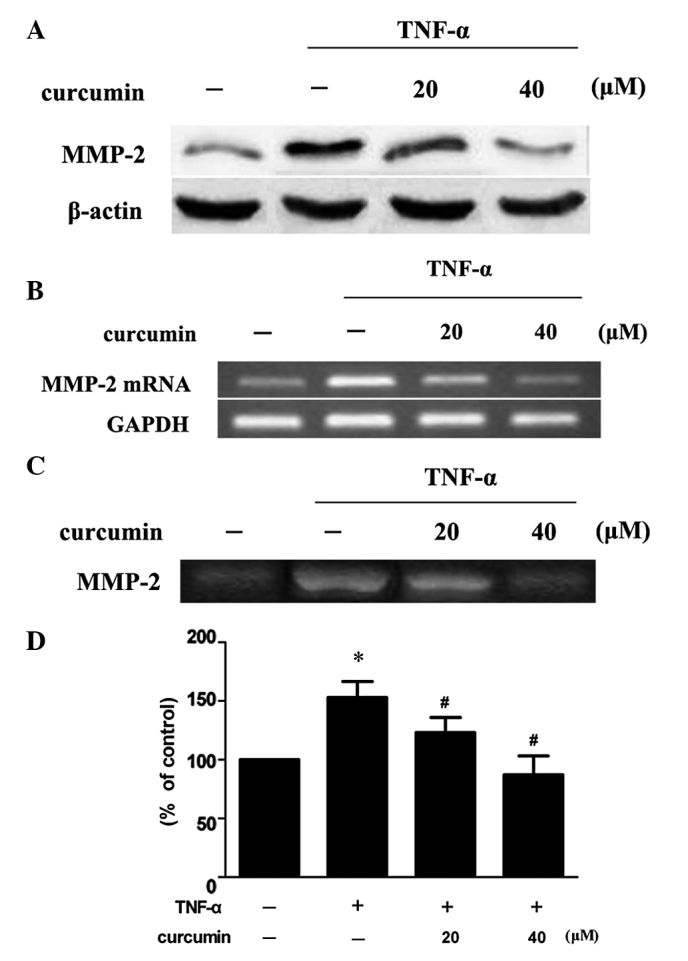
Curcumin inhibits TNF-α-induced MMP-2 expression and activity in VSMCs. VSMCs were pretreated with curcumin (20 or 40 μM) for 1 h and exposed to 100 ng/ml TNF-α for an additional 23 h. Following treatment, the (A) protein expression, (B) mRNA expression and (C) activity levels of MMP-2 were assessed by western blot analysis, RT-PCR and gelatin zymography, respectively. (D) Densitometric analysis was conducted with Image J software to quantify the gelatin zymography data. ^*^P<0.05, vs. control group; ^#^P<0.05, vs. TNF-α + curcumin group. Data are expressed as the mean ± standard deviation of three independent experiments. VSMCs, vascular smooth muscle cells; TNF, tumor necrosis factor; MMP, matrix metalloproteinase; RT-PCR, reverse transcription polymerase chain reaction.

VSMCs were treated with 100 ng/ml TNF-α in the presence or absence of various concentrations of curcumin. Compared with TNF-α alone, 20 and 40 μM curcumin significantly reduced MMP-2 expression and activity levels (P<0.05; Fig. 3). Treatment with 40 μM curcumin was more effective at decreasing the levels of protein expression and activity of MMP-2 when compared with 20 μM curcumin treatment.

### Curcumin suppresses TNF-α-induced MMP-2 expression in VSMCs via the nuclear factor (NF)-κB pathway

**Figure 4:**
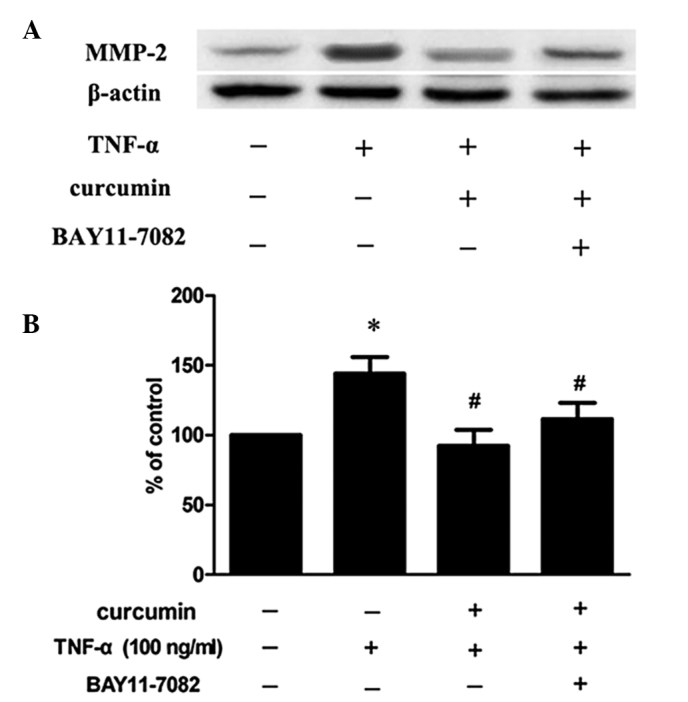
Curcumin suppresses TNF-α-induced MMP-2 expression. (A) VSMCs were pretreated with 40 μM curcumin and 10 μM BAY11-7082 for 1 h and exposed to 100 ng/ml TNF-α for an additional 23 h. Following treatment, the protein expression levels of MMP-2 were determined by western blot analysis. (B) Densitometric analysis was conducted with Image J software to quantify the western blot data. ^*^P<0.05, vs. control group; ^#^P<0.05, vs. TNF-α-treated group. Data are expressed as the mean ± standard deviation of three independent experiments. VSMCs, vascular smooth muscle cells; TNF, tumor necrosis factor; MMP, matrix metalloproteinase.

**Figure 5:**
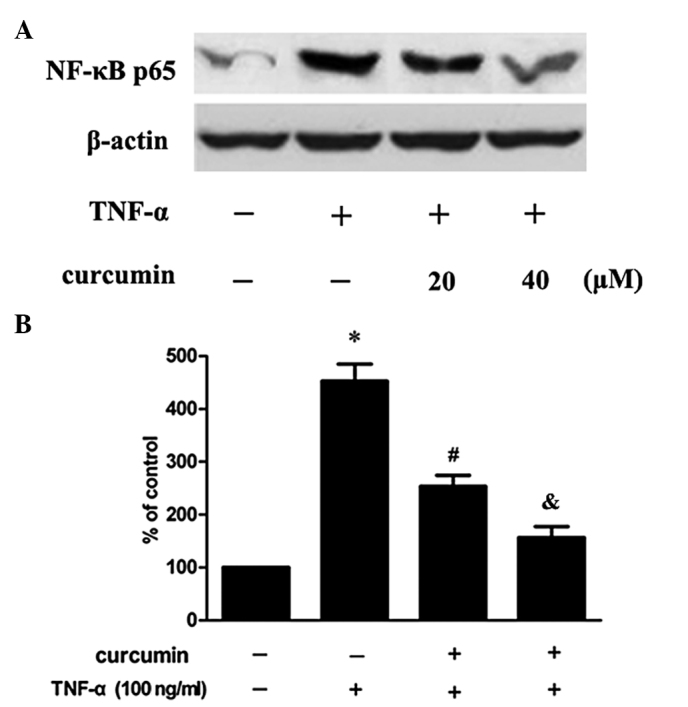
Effects of curcumin on TNF-α-induced NF-κB p65 expression. (A) VSMCs were pretreated with curcumin for 1 h and exposed to 100 ng/ml TNF-α for an additional 23 h. Following treatment, NF-κB p65 expression levels were determined by western blot analysis. (B) Densitometric analysis was conducted with Image J software to quantify the western blot analysis data. ^*^P<0.05, vs. control group; ^#^P<0.05, vs. TNF-α-treated group; ^&^P<0.05, vs. TNF-α + curcumin group. Data are expressed as the mean ± standard deviation of three independent experiments. VSMCs, vascular smooth muscle cells; TNF, tumor necrosis factor; NF, nuclear factor.

In order to further investigate whether the NF-κB signaling pathway is involved in the inhibitory effect of curcumin on the TNF-α-induced expression of MMP-2, cells were pretreated with an inhibitor of NF-κB (BAY11–7082). The results revealed that the inhibitory effect was partly eliminated (Fig. 4). In addition, the effect of curcumin on the p65 subunit, induced by TNF-α, was also determined. As a result, TNF-α was found to increase the expression of the p65 subunit in the nucleus, but this increase was inhibited when the cells had been preincubated with curcumin (Fig. 5).

### Curcumin prevents TNF-α-induced ROS generation

**Figure 6:**
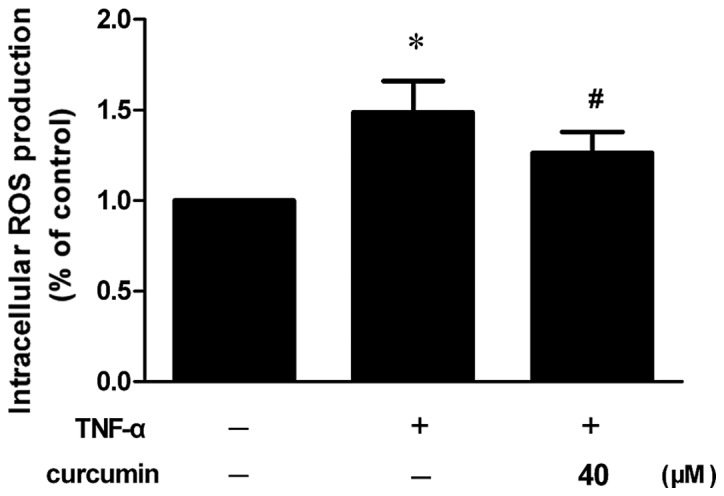
Curcumin prevents TNF-α-induced ROS generation in VSMCs. VSMCs were pretreated with 40 μM curcumin for 1 h and induced by 100 ng/ml TNF-α for 23 h. ^*^P<0.05, vs. control group; ^#^P<0.05, vs. TNF-α-treated group. Data are expressed as the mean ± standard deviation of three independent experiments. VSMCs, vascular smooth muscle cells; TNF, tumor necrosis factor; ROS, reactive oxygen species.

Increased ROS generation was observed in VSMCs that had been stimulated with TNF-α, whereas the inhibitory effect was significantly blocked by pretreatment with curcumin (Fig. 6; P<0.05).

## Discussion

VSMC migration evidently plays a critical role in the pathophysiology of several prominent cardiovascular disease states, including atherosclerosis and restenosis (12,13). Previous studies have shown that the MMP system may be a potential therapeutic target for the treatment of restenosis or atherosclerosis since the MMP system plays a role in VSMC migration and neointima formation following vascular injury (14). In the present study, the inhibitory effect of curcumin on TNF-α-induced VSMC migration was investigated, as well as the possible mechanisms involved. Curcumin was found to inhibit the TNF-α-stimulated migration of VSMCs, which is consistent with a previous study (15). In addition, administration of an MMP inhibitor (GM-6001) was shown to partly diminish the inhibitory effect, indicating that MMP-2 may play an important role in this process.

Accumulating evidence has indicated that gelatinase MMP-2 plays a pivotal role in the initiation and progression of atherosclerotic lesions. MMP-2 is constitutively expressed in VSMCs in normal arteries (9), and MMP-2 expression and activity levels may contribute to the pathogenesis of atherosclerosis by facilitating the migration of VSMCs (16). Therefore, the inhibitory effect of curcumin on the TNF-α-induced expression and activity of MMP-2 was further investigated. Subsequently, curcumin was demonstrated to significantly inhibit TNF-α-induced MMP-2 expression and activity, which indicated that MMP-2 is possibly involved in the inhibitory effect of curcumin on TNF-α-induced cell migration.

TNF-α is one of the major inflammatory cytokines that mediates a wide range of biological responses, including inflammation, infection, injury and apoptosis (17). The effects of TNF-α are initiated by binding to its receptors, which causes the activation of two major transcription factors, AP-1 and NF-κB. This in turn induces the expression of genes involved in inflammatory responses and apoptosis (18). A previous study also demonstrated that inflammatory cytokines, including TNF-α, may induce the expression of the genes that encode MMPs (19). Thus, the present study provides new evidence that TNF-α enhances MMP-2 expression and activity in cultured VSMCs, and to the best of our knowledge, the present study shows for the first time that curcumin significantly inhibits TNF-α-induced MMP-2 expression and activity. Previous studies have indicated that transcriptional regulation involving NF-κB activation has been implicated in the TNF-α-induced activation of VSMCs (20). A key component of MMP expression is the redox-sensitive transcription factor NF-κB (21).

Therefore, to further investigate whether NF-κB contributes to the regulatory effect of curcumin on TNF-α-induced MMP-2 expression and activity, the cells were pretreated with BAY11-7082 (an NF-κB inhibitor) and curcumin prior to the addition of TNF-α. The inhibitory effect was shown to be blocked by BAY11-7082, which indicated that NF-κB is involved in this process.

In unstimulated cells, inactive NF-κB exists as a heterodimeric complex of the subunits, p50 and p65, that are complexed with the inhibitory protein, IκB. Upon activation, phosphorylation of IκB results in its degradation, which is followed by the translocation of the liberated NF-κB to the nucleus where the dimer interacts with regulatory κB elements in promoters and enhancers, thereby controlling gene transcription (22). Consistent with previous observations (19,23,24), the present study also demonstrated that TNF-α activates NF-κB in VSMCs. In addition, a key component of MMP expression is the redox-sensitive transcription factor NF-κB (21). These results indicate that MMP expression, in response to TNF-α, may be mediated by this transcription factor.

In the present study, curcumin was found to reduce TNF-α-induced nuclear translocation of NF-κB p65 in VSMCs. In addition, it was demonstrated that ROS play an essential role in NF-κB activation via proinflammatory cytokines (TNF-α and interleukin-1β) and lipopolysaccharide, two major components of innate immunity (25). The vast majority of studies concerning oxidant-induced NF-κB activation have used H_2_O_2_ as a direct source of ROS. Schreck *et al* (26) were the first to demonstrate that the direct addition of H_2_O_2_ to a culture medium containing a subclone of Jurkat cells (Jurkat JR) resulted in the activation of NF-κB. Excess ROS activate the redox-sensitive transcription factor, NF-κB, resulting in an increase in its activity and expression (27). The activation of NF-κB can be inhibited by antioxidants (28). Thus, the effect of curcumin on TNF-α-induced ROS generation was investigated. Subsequently, TNF-α-induced ROS generation and increased ROS generation was shown to be significantly blocked by pretreatment with curcumin. Therefore, the present study demonstrated that curcumin suppresses TNF-α-stimulated MMP-2 expression and activity in VSMCs via the NF-κB pathway.

In conclusion, curcumin effectively inhibited the TNF-α-induced migration of VSMCs. Levels of ROS production, MMP-2 activation and expression and nuclear translocation of NF-κB p65 were also all reduced by curcumin pretreatment. These results demonstrate that curcumin suppresses TNF-α-induced MMP-2 expression and activity in rat VSMCs via the NF-κB signaling pathway, thereby suppressing cell migration. Therefore, these observations support an emerging role of curcumin as a candidate for the treatment of atherosclerosis. In addition, the ROS/NF-κB pathway may be an additional potential therapeutic target for atherosclerosis-associated diseases.
